# Euglycemic Diabetic Ketoacidosis in a Lung Cancer Patient Using Empagliflozin

**DOI:** 10.1155/2020/7437892

**Published:** 2020-06-30

**Authors:** Jordan Sexe, Chadwick Mayes, Peter Tofts

**Affiliations:** ^1^William Carey University College of Osteopathic Medicine, Hattiesburg, MS, USA; ^2^Internal Medicine Residency Program, Baptist Memorial Hospital-Golden Triangle, Columbus, MS, USA

## Abstract

Diabetic ketoacidosis is a leading cause of morbidity and mortality in diabetic patients, and its diagnosis should be timely and accurate. SGLT2 inhibitors are a new class of antidiabetic medications that increase the renal excretion of glucose. It is thought that increased urinary excretion of glucose will mask hyperglycemia during DKA. This can lead to a delayed diagnosis of DKA and worsen outcomes. In this report, we detail a case of euglycemic DKA in a patient who presented to the Emergency Department meeting criteria for septic shock.

## 1. Introduction

Diabetic ketoacidosis (DKA) is a leading cause of morbidity and mortality in diabetic patients [1]. The diagnostic criteria for DKA are blood glucose > 250 mg/dL, the presence of ketones, serum bicarbonate < 15 mEq/L, arterial pH < 7.3, and a high anion gap metabolic acidosis [2]. Euglycemic DKA is a rare form of DKA manifesting without the characteristic hyperglycemia. Euglycemic DKA is defined with high anion gap metabolic acidosis, presence of urinary and serum ketones, and a blood glucose of <250 mg/dL [3].

In May 2015, the FDA released a statement acknowledging an association between SGLT2 inhibitors and ketoacidosis that may occur with normal blood sugars. The FDA recommended that providers assess for ketoacidosis in patients taking an SGLT2 inhibitor who presented with nausea, vomiting, abdominal pain, fatigue, or shortness of breath [4]. As hyperglycemia in DKA may be absent in patients on SGLT2 inhibitors, DKA diagnosis may be delayed, leading to increased morbidity and mortality.

We describe a case of euglycemic DKA due to SGLT2 inhibitor (empagliflozin) use in a patient who presented to the Emergency Department (ED) with a clinical picture of septic shock. We hope to increase awareness of this misleading disease.

## 2. Case Report

A 58-year-old male with history of stage 4 adenocarcinoma of the lung, type 2 diabetes mellitus, and hypertension was sent to the ED by his oncologist a day after initiating chemotherapy; the patient's chief complaints were generalized weakness and dyspnea. He had a new chemotherapy port and had completed 5 weeks of radiation therapy.

### 2.1. Review of Systems

The patient reported weakness, shortness of breath, decreased appetite, polydipsia, polyuria, and chronic leg swelling. The patient denied fever, headache, chest pain, cough, nausea, vomiting, diarrhea, abdominal pain, dysuria, unexpected weight loss, and sick contacts.

### 2.2. Medications

The patient reported the following medications: empagliflozin 10 mg once daily, insulin aspart 100 U TID, insulin lispro 0-18 U QID, sitagliptin 100 mg once daily, dexamethasone 4 mg QID, alprazolam 0.25 mg TID, famotidine 20 mg BID, omeprazole 20 mg once daily, furosemide 40 mg once daily, lactulose 10 g TID PRN, losartan 25 mg once daily, temazepam 15 mg nightly PRN.

### 2.3. Physical Examination

The patient's initial vitals in the ED were BP 76/50 mmHg, heart rate 103, respiratory rate 20, temperature 97.3°F, and oxygen saturation of 100% on room air. The patient was alert and oriented to person, place, time, and situation. The BMI was 21 kg/m^2^. He appeared lethargic and ill. The cardiovascular exam showed tachycardia with a gallop and S3. The lungs were clear to auscultation bilaterally. The abdomen was soft, nontender, and nondistended and had normal bowel sounds. The skin was warm and dry without rashes. The extremities showed 3 to 4+ pitting edema to the midcalf bilaterally. A portacath was in place over the patient's left anterior chest wall and showed no overlying signs of infection.

### 2.4. Labs

A CBC showed pancytopenia (Table 1). A CMP showed an anion gap of 20 and glucose of 113 g/dL (Table 2). The lactic acid was 1.4 mmol/L, Mg 2.2 mg/dL, procalcitonin 0.05 ng/mL, and proBNP 496 pg/mL. The result of a serum acetone level was large. A urinalysis showed marked glucosuria and ketonuria (Table 3). Swabs for influenzas A and B were both negative. Peripheral blood, portacath, and sputum cultures were drawn. Legionella and Streptococcus pneumoniae urinary antigens were negative. A nasal swab for MRSA was positive.

### 2.5. Imaging

An EKG showed an incomplete right bundle branch block but was otherwise unremarkable. A chest X-ray showed a rounded cavitary lesion in the left lung, a nodularity in the right midlung (both masses consistent with previous lung cancer diagnosis), and a left-sided VAD in good position.

### 2.6. Treatment

*Day 1*: in the ED, the patient received a weight-based fluid bolus of 3 liters of lactated ringers. A norepinephrine drip was started for hypotension. Empiric vancomycin and piperacillin-tazobactam were started in addition to an insulin drip at 6.9 U/hr. The patient was admitted to the ICU. The patient's intravenous (IV) fluids were switched from lactated ringers to D5NaCl 0.45% at 250 cc/hr. The recorded intake was 870 mL and output was approximately 1,400 mL, resulting in a net fluid balance of -530 mL.

*Day 2*: the anion gap closed, and the insulin drip was replaced with a sliding scale and basal insulin per protocol. The K was 3.3 mEq/L and repleted with 40 mEq KCl. Hydrocortisone was added for stress dose steroids. The patient's IV fluids were switched back to lactated ringers. The patient remained on a norepinephrine drip for blood pressure stabilization. The cultures reported no growth to date. The recorded intake was 950 mL and output was 1,780 mL, resulting in a net fluid balance of -830 mL.

*Day 3*: the patient reported feeling better but was transfused 1 unit of packed RBCs for Hb 6.9 g/dL. The K was 2.9 mEq/L and replaced with 40 mEq KCl. The patient's IV fluids were stopped. Midodrine was added while norepinephrine was weaned. The cultures reported no growth to date.

*Day 4*: the patient continued to report feeling better. The K was 3.0 mEq/L and replaced with 40 mEq. The cultures reported no growth to date.

*Day 5*: the patient continued to improve. The Mg was 1.9 mg/dL and replaced with 2 g of MgSO_4_. The K was 2.7 mEq/L and repleted with 40 mEq KCl. The cultures reported no growth to date. The patient was transferred from the ICU to the general medical floor.

*Day 6*: the patient continued to improve clinically, and the electrolytes stabilized. The patient elected for comfort care and was discharged home with hospice.

## 3. Discussion

In this report, we describe a case of euglycemic diabetic ketoacidosis secondary to the use of an SGLT2 inhibitor (empagliflozin). This case was complicated by persistent hypotension in the setting of chemotherapy-induced immunosuppression, which initially suggested a septic shock etiology. This was eventually revealed to be hypovolemic shock secondary to euglycemic DKA. This case is an example of avoiding “anchor bias.”

The top differential diagnoses for this patient were septic shock and euglycemic DKA. Septic shock was possible due to the patient's underlying immunosuppression from radiation and chemotherapy treatment of lung adenocarcinoma in addition to type 2 diabetes. However, due to the use of an SGLT2 inhibitor, low bicarbonate, elevated anion gap, ketonemia, ketonuria, glucosuria, and history of type 2 diabetes, we elected to pursue euglycemic DKA as our principle diagnosis. Further supporting euglycemic DKA were the negative blood, port, and sputum cultures, in addition to clinical improvement with insulin therapy. The patient also reported polyuria and demonstrated adequate urine output, further suggesting a diabetic ketoacidosis versus septic shock.

The SGLT2 transporter reabsorbs up to 90% of glucose in the proximal convoluted tubule [5]. By inhibiting this transporter, there is increased glucosuria and lower plasma glucose. This enhanced glucose clearance is hypothesized to prevent the hyperglycemia during DKA in patients taking an SGLT2 inhibitor [3].

SGLT2 inhibitors are associated with precipitating DKA [6, 7]. It is hypothesized that, since SGLT2 inhibitors decrease plasma glucose levels, there is reduced insulin demand. This promotes an increase in lipolysis and pancreatic alpha cell activity, increasing the level of glucagon and promoting hepatic ketogenesis [6]. In this case, however, based on the timing of the chemotherapy, it is more likely that this stress triggered the DKA rather than the SGLT2 inhibitor.

An alternative explanation for this patient's ketoacidosis could be alcohol. This can present as a euglycemic ketoacidosis but is unlikely. Although the patient reported a history of 6-pack of 12 oz beer per night, he denied alcohol use within the past 6 months. Supporting this were normal GGT, AST, and ALT. In addition, this patient's hypophosphatemia and hypokalemia can both be explained by DKA, pulmonary adenocarcinoma, and/or recent completion of 5 weeks of radiation therapy.

Another explanation for this euglycemic ketoacidosis could be starvation ketosis. However, starvation ketosis rarely presents with a serum bicarbonate < 18 mEq/L [8], while this patient's serum bicarbonate was 13 mEq/L on the day of admission. In addition, starvation ketosis tends to develop after a prolonged fast [9], while this patient only reported 1 day of decreased oral intake. Therefore, this patient's history of type 2 diabetes mellitus, use of an SGLT2 inhibitor, and history of chemotherapy initiation 1 day prior to admission make euglycemic DKA more plausible.

A limitation of this report is that an arterial blood gas (ABG) was not performed. However, in the presence of marked ketonemia, ketonuria, glucosuria, high anion gap, bicarbonate of 13 mEq/L, and history of type 2 diabetes, it can be deduced that this patient was in DKA. An ABG would allow further classification of DKA severity but is not required for diagnosis [1].

## 4. Conclusions

DKA is a serious and potentially fatal complication of diabetes. Early recognition and treatment are critical. SGLT2 inhibitors may mask the classic hyperglycemia during DKA which may delay diagnosis. In this report, we hope to increase awareness of euglycemic DKA.

## Figures and Tables

**Table 1:** The patient's CBC on the day of presentation to the ED revealing pancytopenia consistent with recent completion of 5 weeks of radiation therapy.

CBC	
WBC (K/*μ*L)	2.1
Hgb (g/dL)	8.4
Hct (%)	24.7
Plt (K/*μ*L)	65

**Table 2:** The patient's CMP on the day of presentation to the ED. Of note, the serum blood glucose level was 113 mg/dL. The anion gap was 20.

Comprehensive metabolic panel	
Na (mmol/L)	143
K (mmol/L)	4.4
Cl (mmol/L)	110
HCO_3_ (mmol/L)	13
BUN (mg/dL)	27
Cr (mg/dL)	0.39
Glu (mg/dL)	113
Ca (mg/dL)	7.7
AST (U/L)	19
ALT (U/L)	24
ALP (U/L)	121
T. bili (mg/dL)	0.8
Albumin (g/dL)	1.7
Anion gap	20

**Table 3:** The patient's urinalysis on the day of presentation to the ED.

Urinalysis	
Glucose	>1,000
Ketones	>80
Protein	30
Urobilinogen	2.0
Spec. gravity	1.03
